# Transmission network and phylogenetic analysis reveal older male-centered transmission of CRF01_AE and CRF07_BC in Guangxi, China

**DOI:** 10.1080/22221751.2022.2147023

**Published:** 2022-12-28

**Authors:** Fei Zhang, Yao Yang, Na Liang, Huayue Liang, Yongzheng Chen, Zhaosen Lin, Tongbi Chen, Wenling Tan, Yuan Yang, Rongye Huang, Lin Yao, Fuling Chen, Xingzhen Huang, Li Ye, Hao Liang, Bingyu Liang

**Affiliations:** a Guangxi Key Laboratory of AIDS Prevention and Treatment, School of Public Health, Guangxi Medical University, Nanning, People’s Republic of China; b Collaborative Innovation Centre of Regenerative Medicine and Medical BioResource Development and Application Co-constructed by the Province and Ministry, Life Science Institute, Guangxi Medical University, Nanning, People’s Republic of China; c Qinzhou Center for Disease Control and Prevention, Qinzhou, People’s Republic of China; d Lingshan County Center for Disease Control and Prevention, Qinzhou, People’s Republic of China

**Keywords:** HIV/AIDS, older people, transmission networks, phylogenetic, molecular epidemiology

## Abstract

In China, the number of newly reported HIV infections in older people is increasing rapidly. However, clear information on the impact of older people on HIV transmission is limited. This study aims to reveal the local HIV transmission patterns, especially how older people affect virus transmission. Subtype analysis based on available *pol* sequences obtained from HIV patients revealed that CRF01_AE and CRF08_BC were predominant in patients aged <50 years, whereas CRF01_AE was predominant in older people aged ≥50 years (*χ*^2 ^= 29.299, *P *< 0.001). A total of 25 patients (5.2%, 25/484) were identified with recent HIV infection (RHI). Transmission network analysis found 267 genetically linked individuals forming 55 clusters (2–63 individuals), including 5 large transmission clusters and 12 transmission clusters containing RHI. Bayesian phylogenetic analysis suggested that transmission events in CRF01_AE and CRF07_BC were centred on older males, while transmission events in CRF08_BC were centred on younger males. Multivariable logistic regression analysis showed that older people were more likely to cluster within networks (AOR = 2.303, 95% CI: 1.012–5.241) and that RHI was a significant factor associated with high linkage (AOR = 3.468, 95% CI: 1.315–9.146). This study provides molecular evidence that older males play a central role in the local transmission of CRF01_AE and CRF07_BC in Guangxi. Given the current widespread of CRF01_AE and CRF07_BC in Guangxi, there is a need to recommend HIV screening as part of free national medical examinations for older people to improve early detection, timely treatment, and further reduce second-generation transmission.

## Introduction

The Guangxi Zhuang Autonomous Region (Guangxi) is located in southwestern China, bordering Vietnam. Since the first indigenous case of human immunodeficiency virus (HIV) infection was detected in 1996 [1], the prevalence of acquired immune deficiency syndrome (AIDS) in Guangxi has been increasing dramatically. As of October 2020, the cumulative number of people living with HIV in Guangxi exceeded 97,000, indicating a 39.5% increase since June 2011 (69,548 cases). As a result, Guangxi ranks third with 9.3% of the total reported HIV cases in China, while accounting for less than 4.0% of the national population [2]. The HIV prevalence in Guangxi is three times higher than the national average (1.5% vs. 0.45%) [3]. There are approximately 10,000 newly reported HIV cases in Guangxi each year. Initially, the HIV epidemic in Guangxi was driven by intravenous drug users (IDUs), but the principal route of HIV spread has shifted to sexual transmission (>95%) since 2006 [4]. Due to the hidden nature of sexual transmission and the barriers to intervention, which increase the likelihood of a more diffuse and generalized epidemic [5], HIV poses an increasing threat to the heterosexual population, especially older heterosexuals in rural areas.

As a developing country with a population of more than 1.4 billion, China, like many developed countries, is facing the challenge of an aging population. By 2020, China’s population aged 50 years and older reached 486.58 million, accounting for 34.52% of the total population [6]. The rural population is aging significantly faster than the urban population [6]. Guangxi is a typical province that is “aging before getting wealthy”. As a remote area with relatively slow economic and social development, Guangxi is experiencing an alarming rate of population aging. The demographic shift from middle adults to the elderly has taken only 10 years. According to the results of the 7th national census in 2020, the permanent resident population ≥60 years old in Guangxi accounted for 16.69% of the total population [7]. Lowering the age boundary to 50 years increases this proportion significantly. Older people are at higher risk of various chronic diseases, including AIDS, which places a heavy burden on society and families. This is a severe issue that deserves further attention.

Older HIV patients aged ≥50 years represent a unique group, distinct from the usual sexually active population (15–49 years old) [8]. Recent evidence demonstrated that older people remain sexually active and that an increasing number of older Chinese were living with HIV [9]. A previous meta-analysis noted an HIV prevalence of 1.68% in older people from 2010 to 2018 [10], considerably higher than the 0.058% prevalence found in the general population [11], further emphasizing that older people are of concern. High-risk behaviours, such as having multiple sexual partners and unprotected sex with non-spousal partners, are common among older people [12,13], putting them at higher risk of HIV infection and transmission [14]. The number of newly reported HIV cases among those aged 50 years and older has been increasing year over year [15]. In Guangxi, older people accounted for more than 40% of reported HIV cases in 2013, up from less than 4% in 2000 [4]. In particular, the proportion of older males with HIV has increased to 64.8% [16]. Older people living in rural areas, especially older heterosexual male clients of female sex workers (FSWs) from low-cost commercial sex venues were considered at higher risk of HIV infection [17–19].

Phylogenetic analysis and transmission networks are useful tools that can be used reliably to define closely related clusters that reflect actual transmission [20], and thus guide the development of targeted intervention strategies [21]. Previous studies in China have shown that older people play an crucial role in local HIV transmission with a limited sample size [22], and the studies did not demonstrate viral transmission among older people in large clusters or reveal the impact of new infections in older people on local HIV transmission [23]. Currently, newly reported HIV cases in Guangxi are mainly from the elderly population; however, clear information on the impact of older people on HIV transmission networks and phylogenetics is limited.

Qinzhou city, located in the south of Guangxi, has a serious HIV epidemic. The HIV molecular epidemiological and socio-demographic characteristics of people living with HIV (PLHIV) in Qinzhou are consistent with those of the whole province. Firstly, Qinzhou ranks third among Guangxi’s 14 cities in terms of cumulative HIV/AIDS cases. Secondly, the distribution of HIV subtypes in Qinzhou is consistent with that in Guangxi, mainly Circulating recombinant form (CRF) 01_AE, CRF08_BC and CRF07_BC [24]. Thirdly, the main HIV transmission route in Qinzhou was heterosexual transmission (94.5%) [25], which is comparable with the data in Guangxi (>90%) [4]. Finally, the majority of HIV cases reported in Qinzhou were male (74.2%) and elderly (53.1%) [24], which is also consistent with the situation in Guangxi [4,16].

This study was designed to reveal the local transmission patterns of HIV in Qinzhou, Guangxi. We also focused specifically on the impact of older people on HIV transmission, aiming to inform the development of targeted intervention strategies.

## Methods

### Study population and data collection

From January 1, 2017 to December 31, 2018, 518 previously and newly reported HIV patients were recruited by convenience sampling in all four administrative counties in Qinzhou city. All subjects in this study were diagnosed between 1999 and 2018, and the majority (67.4%) were newly diagnosed between 2017 and 2018. The inclusion criteria were as follows: (1) at least 16 years old; (2) living with HIV; and (3) no previous history of antiretroviral therapy (ART).

Socio-demographic information including age, gender, ethnicity, education, occupation, marital status, transmission route, high-risk sexual behaviour, history of intravenous drug use, date of diagnosis and current place of residence was obtained through a face-to-face interview. Among these, high-risk sexual behaviour was defined as having a homosexual, commercial, or casual sexual partner. The time from diagnosis to sampling was calculated for each participant. Venous blood was extracted, and the plasma was separated and stored at −80°C. After sampling, all participants were included in treatment. Written informed consent was obtained from all participants. This study was reviewed and approved by the Human Research Committee of Guangxi Medical University (No. 20170228-21).

### The maxim HIV-1 LAg-Avidity EIA test

To identify individuals who may have been recently infected at the time of sample collection, an HIV-1 Limiting Antigen Avidity (LAg-Avidity) Enzyme Immunosorbent Assay (EIA) Test (Cat. No. 92001, Maxim Biomedical, Rockville, MD, United States) was performed for samples with less than one year between confirmation of HIV infection and sampling (*n* = 321). A single initial screening test was performed on all plasma samples. Then, a conformation test was performed in triplicate on specimens with an initial screening normalized optical density (ODn) ≤ 2.0. The threshold value for ODn was set at 1.5, corresponding to an average seroconversion of 130 days [26]. Recent HIV infection (RHI) was determined if the median ODn value of the triplicate confirmatory tests was ≤1.5; otherwise, chronic HIV infection (CHI) was determined [27].

### HIV nucleic acid extraction, amplification and sequencing

HIV RNA was extracted using an automated nucleic acid extraction machine (NP968-S system) and the Tianlong RNA extraction Kit (Tianlong, Xian, China) according to the manufacturer’s standard protocol. When the initial extraction was unsuccessful, RNA was extracted again using the High Pure Viral RNA Kit (Roche, Germany). The RNA was then subjected directly to nested polymerase chain reaction (PCR) with the Prime Script One Step RT–PCR Kit (Takara, Dalian, China) to generate *pol* gene fragments (HXB2: 2253-3464) as previously described [28]. Amplification products from PCR-positive samples were purified and sequenced. The chromatogram data were cleaned and assembled using Sequencher v5.4.6 (Gene Codes, Ann Arbor, MI). Only sequences over 900 nucleotides were retained in our analysis, because network inference for shorter sequences is inaccurate [29,30].

### HIV subtype analysis

Firstly, sequences with a proportion of mixed bases greater than 5% were removed using the online tool Quality Control in the Los Alamos National Laboratory HIV Database (hereafter referred to as the HIV Database, https://www.hiv.lanl.gov). The sequences obtained were aligned with a reference dataset downloaded from the HIV Database using the online tool HIV Align and edited manually using BioEdit v7.0. Initial subtype analysis was performed with an approximately maximum likelihood (ML) phylogenetic tree constructed using the general time-reversible substitution (GTR) model in FastTree v2.2.10 and Figtree v1.4.3. Sequences that clustered with the reference sequence and with bootstra*p* values ≥70% were identified as the same subtype as the reference sequence. Sequences that could not be determined by the initial subtype analysis were identified using the online tool HIV BLAST. After these two rounds of subtype analysis, sequences that still could not be determined were defined as unique recombinant forms (URFs).

### HIV transmission network inference

HIV transmission networks were defined by the genetic distance (GD) matrix approach and constructed using the HIV-TRAnsmission Cluster Engine (HIV-TRACE) [31], which has been widely used to construct molecular networks. We performed sensitivity analysis for different GD thresholds (ranging from 0.1% to 2.5%) to determine the optimal threshold (i.e. the GD when the maximum number of transmission clusters is detected). Finally, putative transmission links for the Tamura-Nei 93 GD ≤1.8% were inferred. Each cluster was sorted by the number of nodes in the cluster from highest to lowest and then numbered in ascending order. Large transmission clusters were defined as those containing 10 or more patients. Transmission clusters containing RHI were focused on to determine the characteristics of clusters where recent transmission event existed and the characteristics of RHI. We also ranked the degrees of all nodes within the network, and defined nodes with degrees greater than or equal to the upper quartile (i.e. degree ≥7) as having high linkage. Nodes with degrees ≥15 were defined as high-degree individuals (i.e. individuals with a high risk of transmission).

### Bayesian phylogenetic analysis

To better understand the evolutionary history of HIV in Guangxi, we downloaded 418, 82, and 112 reference sequences from the HIV database for CRF01_AE, CRF08_BC and CRF07_BC, respectively. After deduplication, only reference sequences containing the sampling dates necessary for Bayesian phylogenetic analysis were included. A total of 1,079 sequences were obtained, including 675 CRF01_AE strains, 244 CRF08_BC strains, and 160 CRF07_BC strains (Table S1). Of which, 43.3% (467/1,079) were collected in Qinzhou during 2017–2018, others were downloaded from the HIV Database. Three sub-datasets were created based on the major HIV subtypes.

To reveal the role of older people in HIV transmission events in more detail, we still used 50 years as the age cut-off for older people and defined the following four age-gender subgroups for the local subjects: older male (OM), older female (OF), younger male (YM), and younger female (YF). Temporal signals were evaluated in TempEst v1.5.3 [32], and Bayesian inference was performed in BEAST v1.10.5 using the Skygrid model with an uncorrelated lognormal relaxed clock under the general time reversible (GTR) substitution model. Each Markov Chain Monte Carlo (MCMC) iteration for CRF01_AE, CRF08_BC, and CRF07_BC was run with 600, 400 and 200 million states. Every 1,000 iterations were sampled and the first 10% were discarded as burn-in. Convergence defined as an effective sample size (ESS) ≥ 200, was determined in Tracer v.1.7.2 [33]. A Bayesian Stochastic Search Variable Selection (BSSVS) procedure was used to determine the relationship between subgroups [34]. And a robust counting (Markov jumps) approach was used to calculate the expected number of viral migrations [35]. Statistical support was measured using Bayes Factors (BF) and posterior probability calculated by SpreaD3 v0.9.6 [36]. Only results with BF ≥3 and posterior probability support ≥0.9 were further discussed [37].

### Statistical analysis

Descriptive statistics were performed on socio-demographic parameters comparing patients in transmission clusters, patients in transmission clusters containing RHI, patients in large transmission clusters, and patients with high linkage. Univariable and multivariable logistic regression models were applied to identify factors associated with clustering and high linkage. Multicollinearity between the independent variables was excluded using variance inflation factors (VIF, <5) and correlation coefficients (<0.8). The crude odds ratios (COR), adjusted odds ratios (AOR), and 95% confidence intervals (CI) were calculated. The Chi-square test or Fisher’s exact test was used to compare the characteristics of patients with RHI or CHI in the network. All statistical analyses were performed using IBM SPSS Statistics v26.0 (IBM Corp., Armonk, NY, USA). *P* values were two-sided with a significance level of 0.05.

## Results

### Study population

Of the 518 plasma samples collected from Qinzhou (Figure S1a), 93.4% (484/518) were sequenced successfully and included in the follow-up analysis. According to the Chinese HIV/AIDS case reporting system, the total number of newly reported HIV/AIDS cases in Qinzhou was 481 from 2017 to 2018. Of them, we included 326 cases in our analysis, accounted for 67.7% (326/481) of the total number of newly reported HIV/AIDS cases in Qinzhou during the study period. The sampling depth meet the requirements (60%) for transmission network analysis recommended by the Chinese Center for Disease Control and Prevention (CDC).

As shown in Table 1, we found that 72.7% of the participants were male (352/484), and 48.1% (233/484) were 30–49 years old. Participants were mainly Han (90.7%, 439/484). More than half of the participants were farmers (69.2%, 335/484), had ≤6 years of education (54.6%, 264/484), and were married or cohabiting (57.4%, 278/484). Heterosexual contact was the most common transmission route (80.2%, 388/484), followed by intravenous drug use (17.3%, 84/484). Seventy percent (339/484) of patients had an interval of ≤2 years from diagnosis to sampling. A total of 22.3% of participants (108/484) had engaged in high-risk sexual behaviour, and 21.3% (103/484) had a history of intravenous drug use. The majority of participants (95.5%, 462/484) lived in Qinzhou. And a total of 25 patients (5.2%, 25/484) were identified as RHI.

**Table 1.** Socio-demographic characteristics of HIV patients in Guangxi, 2017–2018.

Variables	Study population (*n* = 484)		Patients in transmission cluster (*n* = 267)		Patients in transmission cluster containing RHI (*n* = 157)		Patients in large transmission cluster (*n* = 134)		Patients with high linkage (*n* = 69)	
	*n*	%	*n*	%	*n*	%	*n*	%	*n*	%
Age										
<30 years old	50	10.4	20	7.5	8	5.1	7	5.2	4	5.8
30–49 years old	233	48.1	114	42.7	71	45.2	64	47.8	27	39.1
≥50 years old	201	41.5	133	49.8	78	49.7	63	47.0	38	55.1
Gender										
Male	352	72.7	192	71.9	115	73.2	100	74.6	52	75.4
Female	132	27.3	75	28.1	42	26.8	34	25.4	17	24.6
Ethnicity										
Han	439	90.7	249	93.3	148	94.3	128	95.5	64	92.8
Zhuang	45	9.3	18	6.7	9	5.7	6	4.5	5	7.2
Education										
≤6 years	264	54.6	161	59.6	94	59.9	80	59.7	42	60.9
7–9 years	170	35.1	91	34.1	53	33.8	46	34.3	24	34.8
≥10 years	50	10.3	17	6.3	10	6.3	8	6.0	3	4.3
Occupation										
Farmer	335	69.2	189	70.8	113	72.0	100	74.6	47	68.1
Others	149	30.8	78	29.2	44	28.0	34	25.4	22	31.9
Marital Status										
Unmarried	117	24.2	61	22.8	38	24.2	35	26.1	14	20.3
Married/Cohabiting	278	57.4	157	58.8	96	61.1	79	59.0	43	62.3
Divorced/Widowed	89	18.4	49	18.4	23	14.7	20	14.9	12	17.4
Transmission Route										
HETs	388	80.2	220	82.4	124	79.0	102	76.1	53	76.8
IDUs	84	17.3	43	16.1	30	19.1	29	21.6	13	18.8
Others	12	2.5	4	1.5	3	1.9	3	2.2	3	4.4
HIV Subtype										
CRF01_AE	257	53.1	145	54.3	71	45.3	51	38.1	42	60.9
CRF08_BC	162	33.5	87	32.6	63	40.1	63	47.0	12	17.4
CRF07_BC	48	9.9	29	10.9	23	14.6	20	14.9	15	21.7
Others	17	3.5	6	2.2	0	0.0	0	0.0	0	0.0
Time from Diagnosis to Sampling										
≤2 years	339	70.0	196	73.4	114	72.6	93	69.4	50	72.5
>2 years	145	30.0	71	26.6	43	27.4	41	30.6	19	27.5
Infection Status										
RHI	25	5.2	25	9.4	25	15.9	12	9.0	13	18.8
CHI	459	94.8	242	90.6	132	84.1	122	91.0	56	81.2
High-risk Sexual Behaviour										
Yes	108	22.3	62	23.2	40	25.5	34	25.4	15	21.7
No	376	77.7	205	76.8	117	74.5	100	74.6	54	78.3
History of Intravenous Drug Use										
Yes	103	21.3	53	19.9	37	23.6	35	26.1	15	21.7
No	381	78.7	214	80.1	120	76.4	99	73.9	54	78.3
Current Place of Residence										
Lingshan	192	39.7	102	38.2	71	45.3	67	50.0	29	42.2
Qinbei	106	21.9	63	23.6	31	19.7	28	20.9	11	15.9
Qinnan	104	21.5	56	21.0	36	22.9	27	20.1	23	33.3
Pubei	60	12.4	41	15.4	17	10.8	11	8.2	5	7.2
Outside Qinzhou	22	4.5	5	1.8	2	1.3	1	0.8	1	1.4

Other genotypes include subtype B, C, G, CRF55_01B and unique recombinant form. Other transmission routes include mem who have sex with men (MSM), blood transmission (BLD) and unknown. Abbreviations: ART, antiretroviral therapy; IDUs, intravenous drug users; HETs, heterosexuals; CRF, circulating recombinant form; RHI, recent HIV infection; CHI, chronic HIV infection.

### HIV subtype distribution

Our analysis showed that eight subtypes were prevalent in Qinzhou. CRF01_AE was the predominant subtype, accounting for 53.1% (257/484), followed by CRF08_BC (33.5%, 162/484) and CRF07_BC (9.9%, 48/484) (Figure S1b). In addition, 17 patients were infected with other subtypes, including subtype B (*n* = 1), C (*n* = 1), G (*n* = 1), CRF55_01B (*n* = 7) and URFs (*n* = 7). There was a significant difference in the composition ratio of subtypes between the ≥50-year-old and the <50-year-old groups (*χ*^2^*^ ^*= 29.299, *P *< 0.001). Older people aged ≥50 years were predominantly infected with CRF01_AE (62.7%, 126/201), followed by CRF08_BC (21.9%, 44/201), CRF07_BC (13.9%, 28/201) and other subtypes (1.5%, 3/201). However, CRF01_AE (46.3%, 131/283) and CRF08_BC (41.7%, 118/283) predominated in a similar proportion of patients younger than 50 years, followed by CRF07_BC (7.1%, 20/283) and other subtypes (4.9%, 14/283) (Figure S1c).

### Identification of HIV transmission clusters

**Figure 1.**
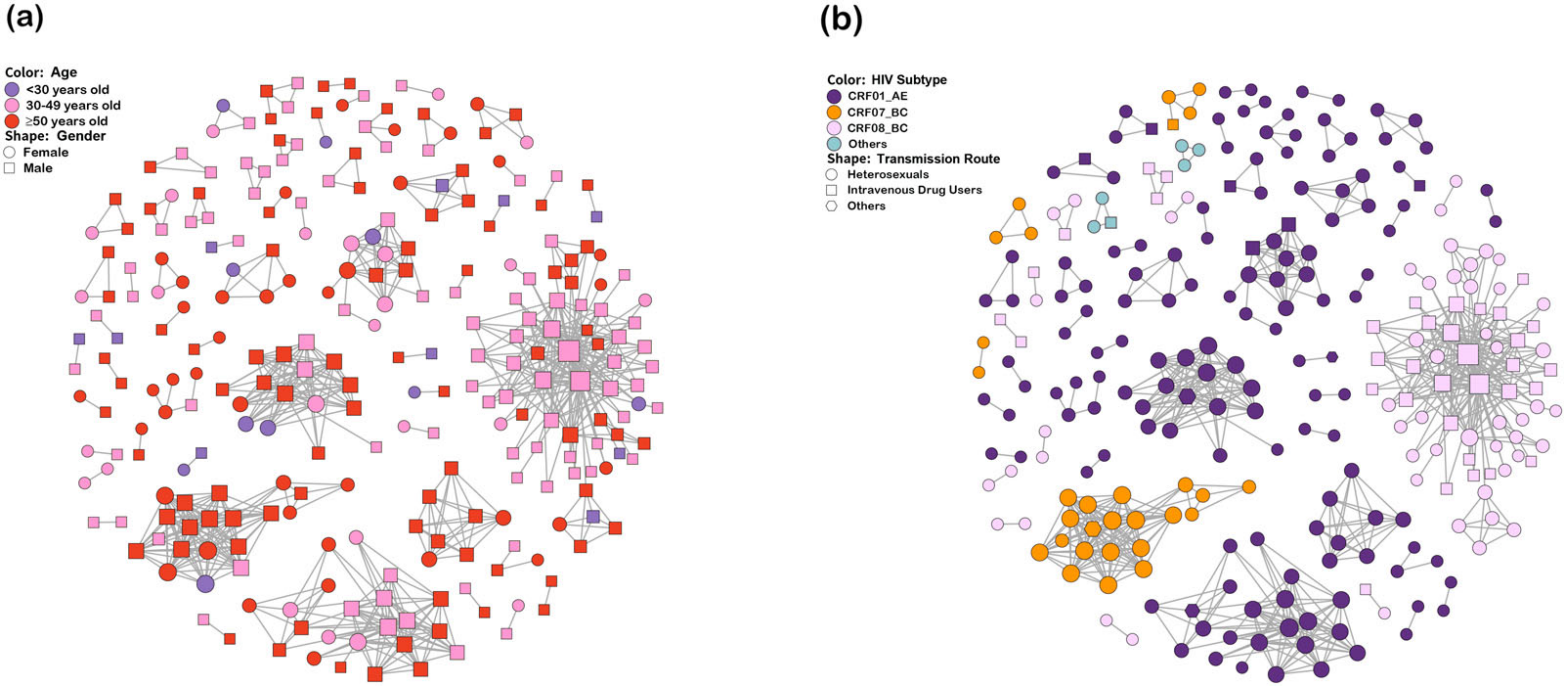
HIV Transmission network in Guangxi. Nodes indicate HIV patients or sequences. The size of a node is proportional to its degree. Edges (i.e. links) represent genetic linkage (≤0.018 substitutions/site). Colours indicate different age groups in (a) and HIV subtypes in (b). Shapes indicate gender in (a) and different transmission routes in (b). Other genotypes include subtype B, C, G, CRF55_01B and unique recombinant form. Other transmission routes include mem who have sex with men (MSM), blood transmission (BLD), and unknown.

Sensitivity analysis of GD thresholds ranging from 0.1% to 2.5% indicated that a threshold of 1.8% was optimal for detecting transmission clusters (Figure S2). The transmission network analysis then revealed that 267 (55.2%) genetically linked individuals formed 55 clusters (2–63 individuals) (Table 1 and Figure 1). Of these, 71.9% (192/267) were male and 28.1% (75/267) were female. The proportion of clustered individuals was 7.5% (20/267) for patients aged <30 years, 42.7% (114/267) for patients aged 30–49 years, and 49.8% (133/267) for patients aged ≥50 years. Four HIV subtypes, including CRF01_AE, CRF08_BC, CRF07_BC and URFs, clustered independently. Similarly, heterosexual contact (82.4%, 220/267) was the main transmission route within the network, followed by intravenous drug use (16.1%, 43/267). The largest cluster was dominated by heterosexuals (HETs) (57.1%, 36/63) and IDUs (42.9%, 27/63), while other clusters were dominated by one or the other. The characteristics of different clusters and non-clustered individuals are shown in Table S2.

### Large transmission clusters

**Figure 2.**
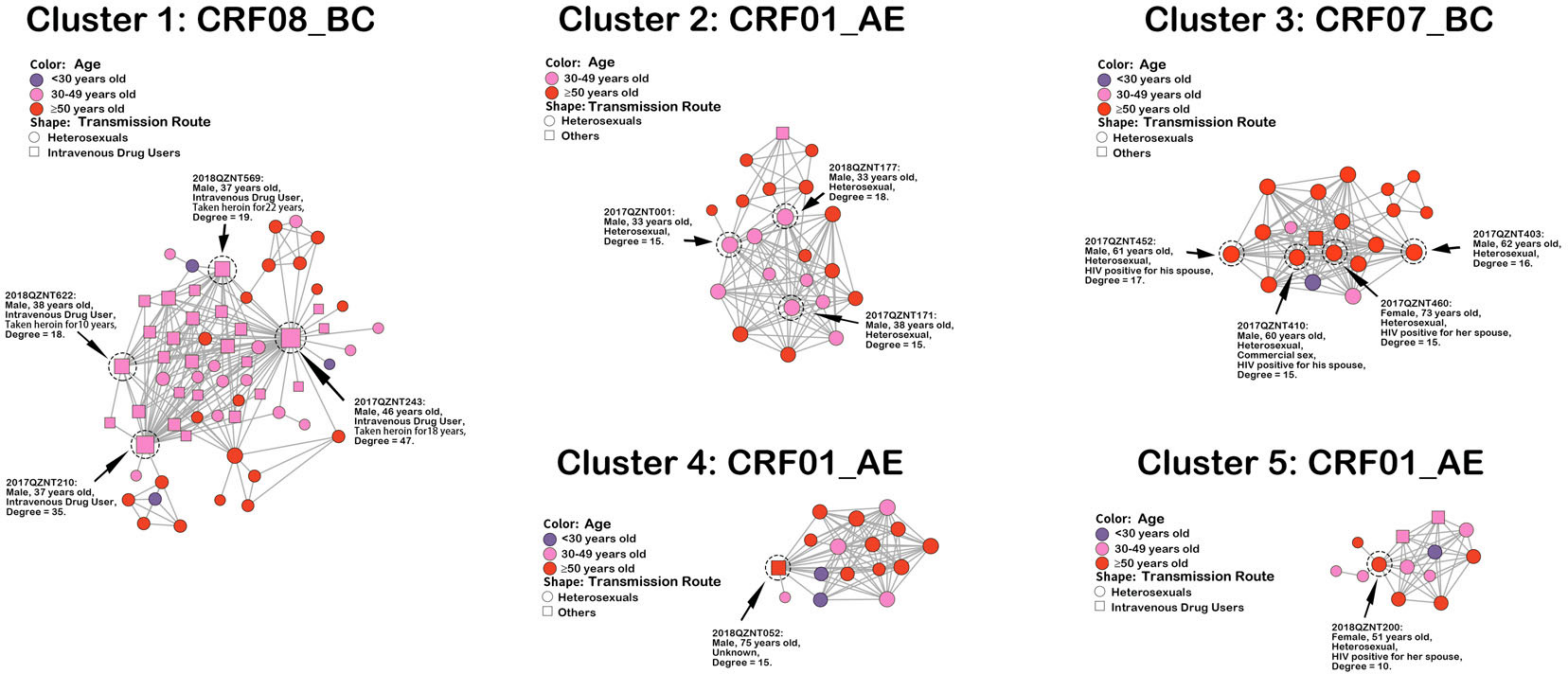
Large transmission clusters. Nodes indicate patients or sequences. The size of a node is proportional to its degree. Edges (i.e. links) represent genetic linkage (≤0.018 substitutions/site). Colours indicate different age groups. Shapes indicate different transmission routes. Nodes with degree ≥15 (i.e. individuals at high risk of HIV transmission) are circled with black dashed lines and indicated by black arrows, except for one node in cluster five with degree  = 10.

We identified five large transmission clusters, including one CRF08_BC cluster, one CRF07_BC cluster and three CRF01_AE clusters (Figure 2). The largest cluster (i.e. cluster one) consisted of HETs (57.1%, 36/63) and IDUs (42.9%, 27/63), all subtyped as CRF08_BC. The high-degree individuals in cluster one were all IDUs who were long-term heroin users (10–22 years). The other four large transmission clusters were predominantly HETs, with 54.5% (12/22), 85.0% (17/20), 62.5% (10/16) and 38.5% (5/13) older people in cluster 2–5, respectively. High-degree individuals in cluster two were HETs aged 30–49 years, and high-degree individuals in clusters three and four were HETs aged ≥50 years. Of note, three high-degree individuals in cluster three reported that their spouses were HIV positive, and one of them also reported a history of commercial sex. A 51-year-old female patient in cluster five, degree 10, also reported her husband as HIV positive. The socio-demographic characteristics of all high-degree individuals are presented in Table S3.

### Transmission clusters containing RHI

**Figure 3.**
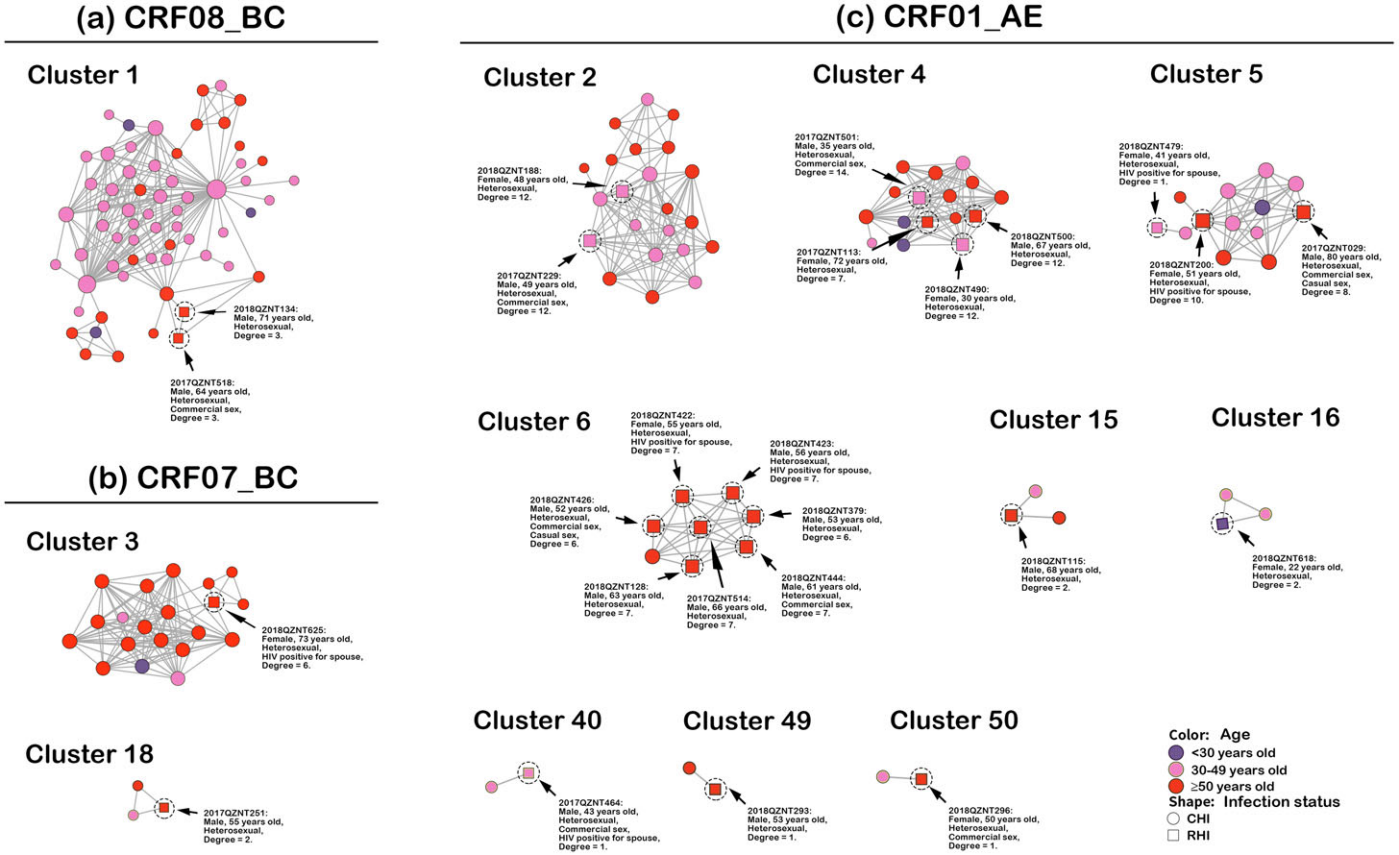
Transmission clusters containing RHI. Nodes indicate patients or sequences. The size of a node is proportional to its degree. Edges (i.e. links) represent genetic linkage (≤0.018 substitutions/site). Colours indicate different age groups. Shapes indicate HIV infection status. Recently infected individuals are circled with black dashed lines and indicated by black arrows. Clusters are arranged by different HIV subtypes. Abbreviations: CRF, circulating recombinant form; CHI, chronic HIV infection; RHI, recent HIV infection.

The transmission network identified 12 transmission clusters containing RHI, including one CRF08_BC cluster, two CRF07_BC clusters and nine CRF01_AE clusters (Figure 3). Individuals with RHI in clusters 1, 3, 6, 15, 18, 49 and 50 were all ≥50 years old, whereas patients with RHI in cluster 2 and 40 were all 30–49 years old. RHI patients in cluster four and five were all aged ≥30 years. A 22-year-old female in cluster 16 was also confirmed with RHI. In this study, 18 of the 25 RHI patients were from the older population, accounting for 72.0%. Notably, all RHI patients identified in this study acquired HIV through HETs. Eight of the 25 RHI patients reported having had commercial sex, and 2 of them reported having had casual sex. Additional socio-demographic and epidemiological characteristics of RHI patients are presented in Table S4.

### Transmission dynamics between age-gender subgroups

**Figure 4.**
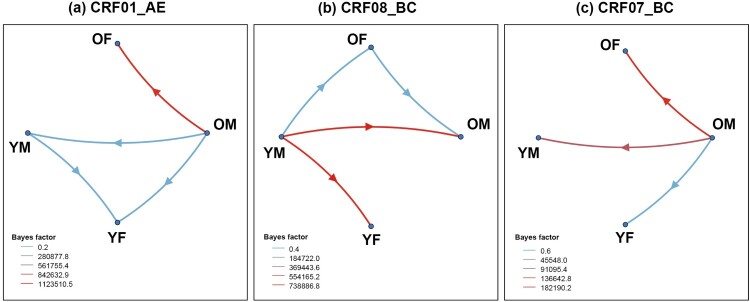
HIV migration events. Well-supported virus dispersal events among age-gender subgroups of (a) CRF01_AE, (b) CRF08_BC, and (c) CRF07_BC. Only results with a Bayes factor (BF) ≥ 3 and posterior probability support ≥0.9 are presented. Arrows indicate the direction of HIV migration events. The colours were chosen to visually distinguish the different level of BF values. Abbreviations: OF, older female; OM, older male; YF, younger female; YM, younger male.

Bayesian phylogenetic analysis revealed a complex history of viral migration between age-gender subgroups, supporting links between older people (especially older males) and other populations (Figure 4 and Table S5). For CRF01_AE, migration events occurred predominantly in the direction of OM toward YM, accounting for 42.51%, followed by transmission from OM to OF (28.19%), from OM to YF (19.67%), and from YM to YF (9.63%). The migration events of CRF08_BC mainly occurred from YM to OM and YM to YF, accounting for 39.68% and 36.77%, respectively. The transmission from YM to OF (15.50%) and from OM to OF (8.06%) was followed. However, CRF07_BC migration events mainly occurred from OM to YM and OM to OF, accounting for 50.08% and 32.02%, respectively. Transmission from OM to YF accounted for 17.91% of the total CRF07_BC migration events. Overall, we observed that OM were the main source of migration events for CRF01_AE (90.37%) and CRF07_BC (100%), and OF and YM were the corresponding destinations. Moreover, YM were the main source of CRF08_BC migration events (91.94%), and OM and YF were the corresponding destinations.

### Factors associated with clustering and high linkages

There was no multicollinearity among the independent variables included in this study. Multivariable logistic regression analysis found that age, ethnicity, education and current place of residence were the most significant factors associated with being part of a transmission cluster (Table 2). Patients aged ≥50 years were more likely to cluster within the network (AOR = 2.303, 95% CI: 1.012–5.241). Additionally, among older people, 66.9% of the linkages were with the older population, and 26.7% were with patients aged 30–49 years (Figure S3a). Individuals with Han ethnicity (AOR = 3.089, 95% CI: 1.381–6.909) and those with a lower education level (≤6 years: AOR = 2.508, 95% CI: 1.190–5.287; 7–9 years: AOR = 2.311, 95% CI: 1.099–4.858) were more likely to cluster. Patients living in Qinnan District (AOR = 0.438, 95% CI: 0.210–0.915) and outside Qinzhou (AOR = 0.137, 95% CI: 0.041–0.452) were less likely to cluster than those living in Pubei County. The HIV transmission pattern in Qinzhou was predominantly intra-regional, with a low rate of inter-regional transmission (Figure S3b). Although RHI was not associated with clustering, all RHI patients were part of the transmission network, and 64.1% of their connections were to CHI patients, while 35.9% were to RHI patients (Figure S3c).

**Table 2.** Factors associated with clustering among HIV patients in Guangxi, 2017–2018.

Variables	Study Population (*n*)	Patient in transmission cluster [*n* (%)]	COR (95% CI)	*P* value	AOR (95% CI)	*P* value
**Age**						
<30 years old	50	20 (40.0)	1		1	
30–49 years old	233	114 (48.9)	1.432 (0.772–2.675)	0.253	1.258 (0.600–2.637)	0.544
≥50 years old	201	133 (66.2)	2.934 (1.552–5.546)	**0**.**001**	2.303 (1.012–5.241)	**0**.**047**
**Gender**						
Male	352	192 (54.5)	1		1	
Female	132	75 (56.8)	1.096 (0.733–1.641)	0.654	1.401 (0.844–2.325)	0.192
**Ethnicity**						
Zhuang	45	18 (40.0)	1		1	
Han	439	249 (56.7)	1.966 (1.052–3.675)	**0**.**034**	3.089 (1.381–6.909)	**0**.**006**
**Education**						
≥10 years	50	17 (34.0)	1		1	
7–9 years	170	91 (53.5)	2.236 (1.158–4.318)	**0**.**017**	2.311 (1.099–4.858)	**0**.**027**
≤6 years	264	161 (61.0)	2.939 (1.558–5.546)	**0**.**001**	2.508 (1.190–5.287)	**0**.**016**
**Occupation**						
Others	149	78 (52.3)	1		1	
Farmer	335	189 (56.4)	1.178 (0.800–1.736)	0.406	0.872 (0.540–1.407)	0.574
**Marital Status**						
Unmarried	117	61 (52.1)	1		1	
Married/Cohabiting	278	157 (56.5)	1.191 (0.772–1.837)	0.429	0.733 (0.423–1.273)	0.270
Divorced/Widowed	89	49 (55.1)	1.125 (0.647–1.955)	0.677	0.662 (0.334–1.315)	0.239
**Transmission Route**						
Others	12	4 (33.3)	1		1	
IDUs	84	43 (51.2)	2.098 (0.587–7.501)	0.255	1.486 (0.307–7.199)	0.623
HETs	388	220 (56.7)	2.619 (0.776–8.844)	0.121	1.633 (0.426–6.266)	0.475
**HIV Subtype**						
CRF01_AE	257	145 (56.4)	1		1	
CRF08_BC	162	87 (53.7)	0.896 (0.604–1.330)	0.586	1.031 (0.628–1.693)	0.905
CRF07_BC	48	29 (60.4)	1.179 (0.629–2.211)	0.608	1.305 (0.647–2.633)	0.457
Others	17	6 (35.3)	0.421 (0.151–1.174)	0.098	0.545 (0.183–1.626)	0.276
**Time from Diagnosis to Sampling**						
>2 years	145	71 (49.0)	1		1	
≤2 years	339	196 (57.8)	1.429 (0.967–2.111)	0.073	1.244 (0.719–2.152)	0.434
**Infection Status**						
CHI	459	242 (52.7)	1		1	
RHI	25	25 (100.0)	1448586946	0.998	1214111167	0.998
**High-risk Sexual Behaviour**						
No	376	205 (54.5)	1		1	
Yes	108	62 (57.4)	1.124 (0.730–1.732)	0.595	1.192 (0.724–1.963)	0.490
**History of Intravenous Drug Use**						
No	381	214 (56.2)	1		1	
Yes	103	53 (51.5)	0.827 (0.535–1.280)	0.394	1.441 (0.535–3.881)	0.469
**Current Place of Residence**						
Pubei	60	41 (68.3)	1		1	
Lingshan	192	102 (53.1)	0.525 (0.284–0.970)	**0**.**040**	0.512 (0.253–1.035)	0.062
Qinbei	106	63 (59.4)	0.679 (0.348–1.324)	0.256	0.874 (0.403–1.895)	0.733
Qinnan	104	56 (53.8)	0.541 (0.278–1.053)	0.071	0.438 (0.210–0.915)	**0**.**028**
Outside Qinzhou	22	5 (22.7)	0.136 (0.044–0.424)	**0**.**001**	0.137 (0.041–0.452)	**0**.**001**

Other genotypes include subtype B, C, G, CRF55_01B and unique recombinant form. Other transmission routes include mem who have sex with men (MSM), blood transmission (BLD) and unknown. Statistically significant *P* values are indicated in bold. Abbreviations: ART, antiretroviral therapy; COR, crude odds ratios; AOR, adjusted odds ratios; CI, confidence intervals; IDUs, intravenous drug users; HETs, heterosexuals; CRF, circulating recombinant form; RHI, recent HIV infection; CHI, chronic HIV infection.

We defined nodes with a degree ≥7 as those with high linkage. Multivariable logistic regression analysis showed that HIV subtype, infection status and current place of residence were significant factors associated with high linkage (Table 3). Compared with CRF01_AE, CRF08_BC (AOR = 0.167, 95% CI: 0.061–0.459) had lower linkage, while CRF07_BC (AOR = 3.635, 95% CI: 1.384–9.548) had higher linkage. Patients living in Lingshan County (AOR = 7.170, 95% CI: 1.999–25.721) and Qinnan District (AOR = 4.824, 95% CI: 1.476–15.762) were more likely to have high linkage than those living in Pubei County. In addition, RHI patients had a higher degree of linkage within the network (AOR = 3.468, 95% CI: 1.315–9.146). Further analysis revealed that a higher proportion of RHI patients were older than CHI patients (72.0% vs. 47.5%, *χ^2 ^*= 5.431, *P *= 0.020) (Table 4). RHI and CHI patients also differed in terms of transmission route, HIV subtype, time from diagnosis to sampling, history of intravenous drug use and current place of residence.

**Table 3.** Factors associated with high linkage (i.e. degree ≥7) among clustered individuals within networks.

Variables	Patients in transmission cluster (*n*)	Patients with high linkage [*n* (%)]	COR (95% CI)	*P* value	AOR (95% CI)	*P* value
**Age**						
<30 years old	20	4 (20.0)	1		1	
30–49 years old	114	27 (23.7)	1.241 (0.382–4.030)	0.719	1.119 (0.260–4.810)	0.880
≥50 years old	133	38 (28.6)	1.600 (0.502–5.096)	0.426	1.241 (0.265–5.817)	0.784
**Gender**						
Male	192	52 (27.1)	1		1	
Female	75	17 (22.7)	0.789 (0.421–1.478)	0.459	0.522 (0.230–1.180)	0.118
**Ethnicity**						
Zhuang	18	5 (27.8)	1		1	
Han	249	64 (25.7)	0.899 (0.309–2.622)	0.846	0.570 (0.146–2.225)	0.419
**Education**						
≥10 years	17	3 (17.6)	1		1	
7–9 years	91	24 (26.4)	1.672 (0.442–6.329)	0.449	1.532 (0.295–7.967)	0.612
≤6 years	161	42 (26.1)	1.675 (0.458–6.121)	0.435	1.481 (0.294–7.459)	0.634
**Occupation**						
Others	78	22 (28.2)	1		1	
Farmer	189	47 (24.9)	0.843 (0.465–1.525)	0.571	0.965 (0.445–2.096)	0.929
**Marital Status**						
Unmarried	61	14 (23.0)	1		1	
Married/Cohabiting	157	43 (27.4)	1.266 (0.634–2.530)	0.504	1.559 (0.613–3.964)	0.351
Divorced/Widowed	49	12 (24.5)	1.089 (0.450–2.633)	0.850	1.271 (0.398–4.053)	0.686
**Transmission Route**						
Others	4	3 (75.0)	1		1	
IDUs	43	13 (30.2)	0.144 (0.014–1.522)	0.107	0.164 (0.005–5.552)	0.315
HETs	220	53 (24.1)	0.106 (0.011–1.039)	0.054	0.076 (0.004–1.360)	0.080
**HIV Subtype**						
CRF01_AE	145	42 (29.0)	1		1	
CRF08_BC	87	12 (13.8)	0.392 (0.193–0.796)	**0**.**010**	0.167 (0.061–0.459)	**0**.**001**
CRF07_BC	29	15 (51.7)	2.628 (1.167–5.918)	**0**.**020**	3.635 (1.384–9.548)	**0**.**009**
Others	6	0 (0.0)	0 (0)	0.999	0 (0)	0.999
**Time from Diagnosis to Sampling**						
>2 years	71	19 (26.8)	1		1	
≤2 years	196	50 (25.5)	0.937 (0.506–1.735)	0.837	0.965 (0.349–2.668)	0.946
**Infection Status**						
CHI	242	56 (23.1)	1		1	
RHI	25	13 (52.0)	3.598 (1.554–8.331)	**0**.**003**	3.468 (1.315–9.146)	**0**.**012**
**High-risk Sexual Behavior**						
No	205	54 (26.3)	1		1	
Yes	62	15 (24.2)	0.892 (0.462–1.725)	0.735	0.836 (0.376–1.859)	0.660
**History of Intravenous Drug Use**						
No	214	54 (25.2)	1		1	
Yes	53	15 (28.3)	1.170 (0.597–2.292)	0.648	1.446 (0.209–9.987)	0.709
**Current Place of Residence**						
Pubei	41	5 (12.2)	1		1	
Lingshan	102	29 (28.4)	2.860 (1.022–8.009)	**0**.**045**	7.170 (1.999–25.721)	**0**.**003**
Qinbei	63	11 (17.5)	1.523 (0.487–4.759)	0.469	1.806 (0.492–6.625)	0.373
Qinnan	56	23 (41.1)	5.018 (1.710–14.722)	**0**.**003**	4.824 (1.476–15.762)	**0**.**009**
Outside Qinzhou	5	1 (20.0)	1.800 (0.166–19.500)	0.629	1.501 (0.083–27.284)	0.784

Other genotypes include subtype B, C, G, CRF55_01B and unique recombinant form. Other transmission routes include mem who have sex with men (MSM), blood transmission (BLD) and unknown. Statistically significant *P* values are indicated in bold. Abbreviations: COR, crude odds ratios; AOR, adjusted odds ratios; CI, confidence intervals; IDUs, intravenous drug users; HETs, heterosexuals; CRF, circulating recombinant form; RHI, recent HIV infection; CHI, chronic HIV infection.

**Table 4.** Differences between RHI and CHI patients among clustered individuals within networks

Variables	Patients in transmission cluster (*n*)	RHI patients		CHI patients		*χ*^2^	*P* value
		*n*	%	*n*	%		
**Age**						**5.431**	**0.020**
<50 years old	134	7	28.0	127	52.5		
≥50 years old	133	18	72.0	115	47.5		
**Gender**						0.854	0.355
Male	192	16	64.0	176	72.7		
Female	75	9	36.0	66	27.3		
**Ethnicity**						2.311	0.128*
Han	249	21	84.0	228	94.2		
Zhuang	18	4	16.0	14	5.8		
**Education**						2.873	0.646
≤6 years	161	17	68.0	142	58.7		
7–9 years	91	7	28.0	84	34.7		
≥10 years	17	1	4.0	16	6.6		
**Occupation**						1.552	0.213
Farmer	189	15	60.0	174	71.9		
Others	78	10	40.0	68	28.1		
**Marital Status**						1.858	0.395
Unmarried	61	3	12.0	58	24.0		
Married/Cohabiting	157	17	68.0	140	57.8		
Divorced/Widowed	49	5	20.0	44	18.2		
**Transmission Route**						**6.500**	0.036**
HETs	220	25	100.0	195	80.6		
IDUs	43	0	0.0	43	17.8		
Others	4	0	0.0	4	1.6		
**HIV Subtype**						**10.222**	0.012**
CRF01_AE	145	21	84.0	124	51.2		
CRF08_BC	87	2	8.0	85	35.1		
CRF07_BC	29	2	8.0	27	11.2		
Others	6	0	0.0	6	2.5		
**Time from Diagnosis to Sampling**						**9.992**	**0.002**
≤2 years	196	25	100.0	171	70.7		
>2 years	71	0	0.0	71	29.3		
**High-risk Sexual Behavior**						1.192	0.275
Yes	62	8	32.0	54	22.3		
No	205	17	68.0	188	77.7		
**History of Intravenous Drug Use**						5.524	0.019*
Yes	53	0	0.0	53	21.9		
No	214	25	100.0	189	78.1		
**Current Place of Residence**						**10.370**	0.027**
Lingshan	102	5	20.0	97	40.1		
Qinbei	63	5	20.0	58	24.0		
Qinnan	56	12	48.0	44	18.2		
Pubei	41	3	12.0	38	15.7		
Outside Qinzhou	5	0	0.0	5	2.0		

Other genotypes include subtype B, C, G, CRF55_01B and unique recombinant form. Other transmission routes include mem who have sex with men (MSM), blood transmission (BLD) and unknown. * indicates Yates’s correction for continuity. ** indicates Fisher’s exact test. Statistically significant *P* values are indicated in bold. Abbreviations: IDUs, intravenous drug users; HETs, heterosexuals; CRF, circulating recombinant form; RHI, recent HIV infection; CHI, chronic HIV infection.

## Discussion

This study is the first to explore the differential impact of older people on the network connection and virus transmission of the three major CRFs through an HIV molecular epidemiological survey in Guangxi. Evidence from network and phylogenetic analysis suggests that older people, especially OM, contribute to the rapid, sustained and complex HIV epidemic in Qinzhou, informing the development of targeted prevention and intervention strategies.

In accordance with a previous study, the most prevalent HIV subtypes in Qinzhou are still dominated by CRF01_AE, CRF08_BC and CRF07_BC [25]. This may reflect the ongoing and sustained transmission of HIV derived from already-circulating local strains [38]. Moreover, the prevalence of CRF01_AE and CRF07_BC was higher in older people than in patients under 50 years of age. This discrepancy may be related to the transmission characteristics of different HIV strains. Previous studies in China found an increased prevalence of CRF01_AE and CRF07_BC in sexually transmitted HIV populations [39,40]. Especially in Guangxi, CRF01_AE predominated in the sexual transmission of HIV [41]. Moreover, due to the high proportion of X4 tropism [42,43], CRF01_AE tended to be associated with rapid disease progression and advanced immunodeficiency [44]. Even with combination ART, poor immune recovery was more frequent with CRF01_AE [45]. Older people tend to be in poor health and vulnerable to multiple diseases, and improving health-related quality of life is more important than just prolonging life. Therefore, it is necessary to enhance comprehensive treatment, care services and disease progression monitoring for older HIV patients.

In this study, we found a relatively low rate of new infections (5.2%), which may be associated with the high late diagnosis rate in Guangxi (70.2%) [46]. More critically, the rate of late diagnosis was much higher in patients aged 50 years and older [47]. In this study, 33.7% (163/484) of subjects had been diagnosed for more than 1 year, and 41.5% (201/484) were aged ≥50 years. Therefore, it is not surprising to find such a low rate of new infections. Our study found a higher linkage for patients with RHI. The most important reason may be that HIV-infected patients are more likely to transmit the virus in the early stage (acute or recent) of infection, when viral loads are high and opportunities for intervention are limited because they are often unaware of their status [48,49]. Higher linkages were also found in CRF07_BC compared to CRF01_AE, which may be related to the rapid expansion of CRF07_BC in recent years among sexually transmitted populations in China due to its slow disease progression, decreased virulence and enhanced transmissibility [50,51].

We found that older people were more likely to be present in transmission clusters, suggesting that they contribute significantly to local HIV transmission and should be monitored as a priority. The psychological and emotional needs of older people are magnified by the lack of companionship and care, and many of them turn to extramarital sexual relationships (such as with FSWs) for stimulation. There is already genetic evidence of an HIV transmission linkage between older male clients and FSWs [52]. Older male clients of low-cost commercial sex venues in rural Guangxi were at higher risk of HIV infection, especially those without stable sexual partners [18,19]. Additionally, a higher proportion of older male clients have numerous sexual partners and use condoms less frequently, making them an important bridge for HIV transmission from FSWs to low-risk groups [53]. Therefore, access to and use of condoms, as well as screening for HIV infection among older people, especially older males and their sexual partners, should be promoted in the future.

In addition, the sources of transmission events inferred from Bayesian phylogenetic analysis in this study were all male. Specifically, we found that transmission events in CRF01_AE and CRF07_BC were OM-centred, whereas transmission events in CRF08_BC were YM-centred. Previous studies found that CRF01_AE and CRF07_BC were prevalent in sexually transmitted populations [54,55], with CRF01_AE being the most prominent subtype in Guangxi and dominating the HIV epidemic in heterosexuals [41]. In recent years, there has been a rapid increase in the number of HIV infections in older people (mainly male) through sexual contact. As an emerging high-risk group, OM could easily transmit the virus to OF through heterosexual commercial sex, and some of them might transmit HIV to YM through homosexual contact or indirect bridge people. However, CRF08_BC originated in Yunnan Province and was prevalent among both IDUs and heterosexuals [56,57]. In this study, approximately 40.0% of CRF08_BC in Qinzhou occurred among IDUs with a predominantly YM population, and the risk of transmission was high [58,59]. Therefore, CRF08_BC is easily transmitted from the YM population to other subpopulations. For example, YM may transmit the virus to OM through intravenous drug use or bridge people, or to YF through heterosexual contact. These findings indicate that the male population, especially OM, plays a key role in HIV transmission in Guangxi. Improved HIV detection is urgently needed to facilitate early treatment and effective prevention of second-generation transmission.

In this study, a total of five large transmission clusters were identified, with cluster one being the largest. Most of the individuals in this cluster were CHI middle-aged IDUs, and only a few were RHI heterosexual older people. In addition, all of the high-degree individuals were long-term heroin-using IDUs (10–22 years). These results suggested that HIV was still rampant in the middle-aged population in Qinzhou through intravenous drug use and sexual contact, and was gradually being transmitted to older people through sexual contact [57]. The other four large clusters were heterosexual clusters, with most nodes coming from the elderly population. In particular, the high-degree individuals and RHI patients in cluster three were all elderly. Moreover, 40.0% of individuals in this cluster reported that their spouses were HIV positive, indicating a higher risk of HIV transmission among older couples. A cohort study in Tanzania found that sero-discordant couples had much higher rates of HIV seroconversion within marriage than the general population, and that female spouses were at higher risk of acquiring HIV from their husbands [60]. However, with ART, the estimated risk of transmission will be greatly reduced [61]. Therefore, it is extremely essential to increase ART among infected elderly patients.

Patients with RHI are at a higher risk of transmission due to their high viral load in the early stages of infection and therefore they can represent recent transmission events to some extent [49]. The Chinese CDC’s guidelines for HIV Transmission Network recommend that clusters containing RHI should be considered as priority clusters for surveillance and intervention. It is therefore essential to identify RHI in newly diagnosed patients. This study identified 25 RHI patients who had acquired HIV through heterosexual contact, consistent with a previous study [4]. Also, older people accounted for 72.0% of the RHI, validating the fact that they are a high-risk group for new infections [16]. To make matters worse, HIV infections in the elderly population may be underestimated due to lack of motivation to seek testing [62] and late diagnosis [63]. Also, due to the commercial heterosexual sex between older males and FSW [52], aggregated clusters could easily form, which also explained the higher linkage of recently infected patients. Of the 12 transmission clusters containing RHI, cluster six was dominated by recently infected elderly patients, which requires targeted intervention and ongoing monitoring. The fact that RHI patients were linked within the network and associated with high linkage, suggests that RHI patients play a key role in local HIV transmission and should be the primary target of interventions. For example, strengthening treatment to achieve and maintain an undetectable viral load may reduce the risk of sexually transmitted HIV.

This study has several limitations. First, our results represent only the HIV epidemic in Qinzhou. Although new infections in older people are also increasing in different regions, the circumstances may differ from region to region. Therefore, future studies should pay attention to the contribution of older people to HIV transmission in a larger geographic context. Second, due to the shortcomings of cross-sectional studies, we were unable to assess the dynamics of HIV transmission. Long-term observation studies are needed to gain more information on HIV transmission patterns. Third, the participants included in this study were previously or newly reported cases, not newly infected individuals. Also, not all HIV cases could be included in our analysis, which may have led to an incomplete transmission network. Future studies should increase sampling efforts where possible. Finally, the reliability of the relevant statistical analysis may be limited by the small number of RHI identified in this study. We will collect more samples and use a larger sample size in future studies to improve the reliability of the statistical results.

In summary, our study reveals a complex HIV epidemic in Qinzhou, Guangxi. We found evidence that older people play a crucial role in the local transmission of HIV. Older people are more likely to be connected within networks. More importantly, OM was the primary source of transmission events for CRF01_AE and CRF07_BC. This suggests that HIV screening should be integrated into free medical examinations for older people nationwide in order to improve early detection and establish effective links with comprehensive treatment and care services. The government should provide more information materials, such as videos and television commercials, to promote HIV and STD testing among older people who engage in high-risk behaviours. Priority should also be given to identifying transmission hotspots and designing targeted interventions to reduce second-generation transmission.

## Supplementary Material

Supplemental Material
