# Supplementary material for: Transmission network and phylogenetic analysis reveal older male-centered transmission of CRF01_AE and CRF07_BC in Guangxi, China
Source: Emerg Microbes Infect. 2022 Dec 28;12(1):2147023. doi: 10.1080/22221751.2022.2147023 (PMC9809400; doi:10.1080/22221751.2022.2147023)
Supplement: Supplemental Material [file TEMI_A_2147023_SM3865.docx]

Supplementary materials for

**Transmission network and phylogenetic analysis reveal older male-centered transmission of CRF01_AE and CRF07_BC in Guangxi, China**

Fei Zhang^1,2#^, Yao Yang^1#^, Na Liang^1#^, Huayue Liang^1^, Yongzheng Chen^3^, Zhaosen Lin^3^, Tongbi Chen^3^, Wenling Tan^4^, Yuan Yang^2^, Rongye Huang^3^, Lin Yao^4^, Fuling Chen^4^, Xingzhen Huang^4^, Li Ye^1,2^*, Hao Liang^1,2^*, Bingyu Liang^1,2^*

1.Guangxi Key Laboratory of AIDS Prevention and Treatment, School of Public Health, Guangxi Medical University, Nanning 530021, Guangxi, China

2.Collaborative Innovation Centre of Regenerative Medicine and Medical BioResource Development and Application Co-constructed by the Province and Ministry, Life Science Institute, Guangxi Medical University, Nanning 530021, Guangxi, China

3.Qinzhou Center for Disease Control and Prevention, Qinzhou 535000, Guangxi, China

4.Lingshan County Center for Disease Control and Prevention, Qinzhou 535000, Guangxi, China

^#^These authors contributed equally to this paper.

* Corresponding author: **Bingyu Liang**, Guangxi Key Laboratory of AIDS Prevention and Treatment, School of Public Health & Life Science Institute, Guangxi Medical University, Email: [liangbingyu@gxmu.edu.com](mailto:liangbingyu@gxmu.edu.com). **Hao Liang**, Guangxi Key Laboratory of AIDS Prevention and Treatment, School of Public Health & Life Science Institute, Guangxi Medical University, Email: lianghao@gxmu.edu.com. **Li Ye**, Guangxi Key Laboratory of AIDS Prevention and Treatment, School of Public Health & Life Science Institute, Guangxi Medical University, Email: yeli@gxmu.edu.com.


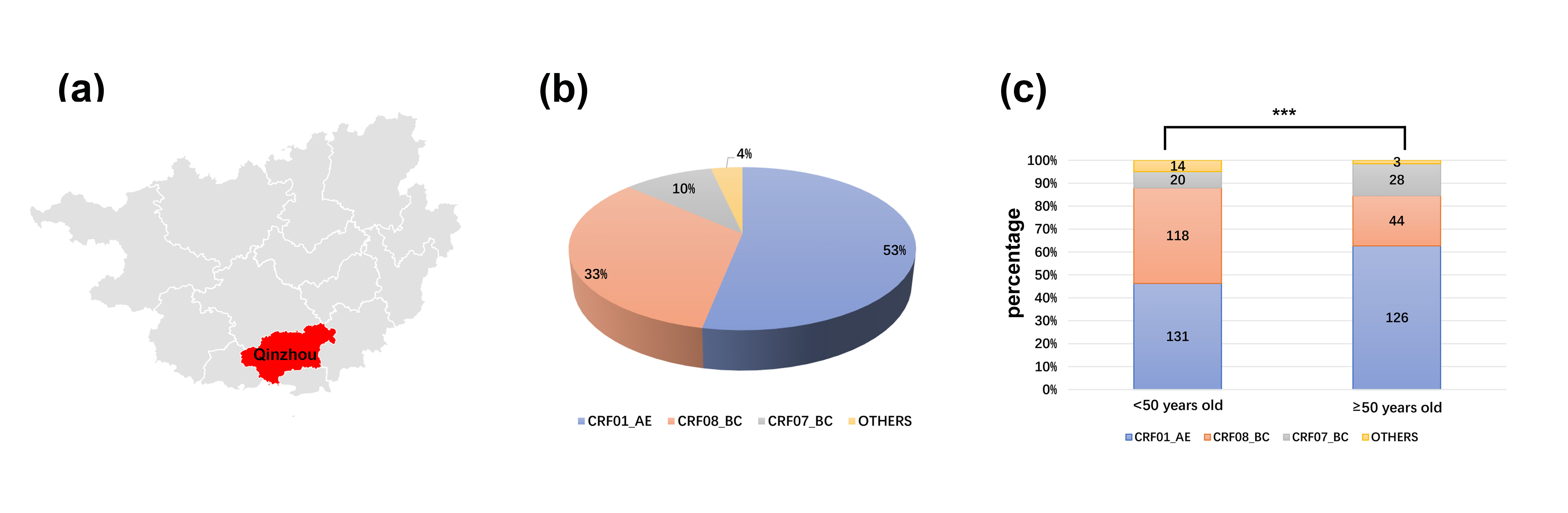


**Figure S1. Distribution of HIV subtypes in Guangxi.** (a) Map of China highlighting Qinzhou, Guangxi. (b) Pie chart representing the HIV subtype distribution. (c) Proportional differences in the composition of HIV subtypes by age group. ***P<0.001. Other genotypes include subtype B, C, G, CRF55_01B and unique recombinant form. Abbreviations: CRF, circulating recombinant form.





**Figure S2 Selection of the optimal genetic distance (GD) threshold.** Solid dotted curves represent the number of clusters at different GD thresholds. Solid square curves represent the number of nodes at different GD thresholds. Solid triangle curves represent the number of links at different GD thresholds. The red dashed line indicates the optimal GD threshold.

**
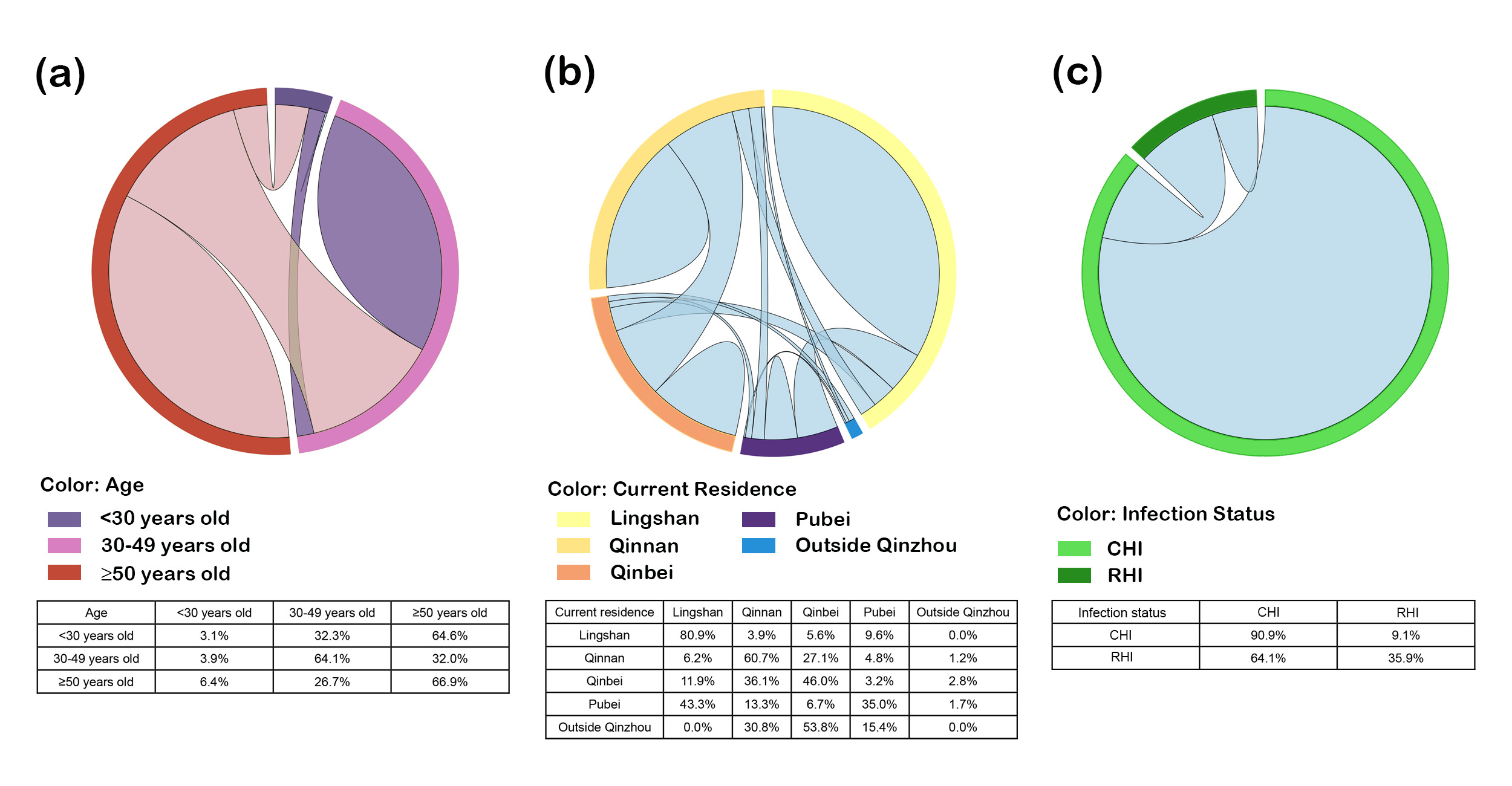
**

**Figure S3 Network attribute relationships.** Correspondence between (a) age, (b) current residence, and (c) recent infection attributes of connected nodes. Abbreviations: CHI, chronic HIV infection; RHI, recent HIV infection.

Table S1 Details of the sampling and diagnostic times for sequences included in this study

| **Variables** | **CRF01_AE dataset** | |  | **CRF08_BC dataset** | |  | **CRF07_BC dataset** | |  | **Other subtypes** |
| --- | --- | --- | --- | --- | --- | --- | --- | --- | --- | --- |
|  | **Local** | **Downloaded** |  | **Local** | **Downloaded** |  | **Local** | **Downloaded** |  | **Local** |
| Total number | 257 | 418 |  | 162 | 82 |  | 48 | 112 |  | 17 |
| Year of sample collection |  |  |  |  |  |  |  |  |  |  |
| Before 2017 | — | 415 |  | — | 82 |  | — | 112 |  | — |
| 2017 | 116 | 1 |  | 60 | 0 |  | 23 | 0 |  | 6 |
| 2018 | 141 | 2 |  | 102 | 0 |  | 25 | 0 |  | 11 |
| Year of patient diagnosis |  |  |  |  |  |  |  |  |  |  |
| Before 2017 | 56 | — |  | 87 | — |  | 12 | — |  | 3 |
| 2017 | 100 | — |  | 33 | — |  | 15 | — |  | 5 |
| 2018 | 101 | — |  | 42 | — |  | 21 | — |  | 9 |

Table S2 Characteristics of different clusters and non-clustered individuals in Guangxi, 2017-2018

| **Cluster ID** | **Cluster Size** | **≥50 years old**  **[n (%)]** | **<50 years old**  **[n (%)]** | **Recent infection**  **[n (%)]** | **Long-term infection**  **[n (%)]** | **Links per node [Mean (Median, IQR)]** | **Genetic distances**  **[Mean (Median, IQR)]** |
| --- | --- | --- | --- | --- | --- | --- | --- |
| 1 | 63 | 19 (30.2) | 44 (69.8) | 2 (3.2) | 61 (96.8) | 5.6 (4.0, 2.0-6.0) | 0.014 (0.016, 0.013-0.017) |
| 2 | 22 | 12 (54.5) | 10 (45.5) | 2 (9.1) | 20 (90.9) | 8.5 (8.5, 4.0-12) | 0.013 (0.014, 0.012-0.015) |
| 3 | 20 | 17 (85.0) | 3 (15.0) | 1 (5.0) | 19 (95.0) | 12.0 (14, 11-14) | 0.012 (0.013, 0.0093-0.014) |
| 4 | 16 | 10 (62.5) | 6 (37.5) | 4 (25.0) | 12 (75.0) | 9.5 (9.5, 7.0-13) | 0.012 (0.012, 0.0093-0.015) |
| 5 | 13 | 5 (38.5) | 8 (61.5) | 3 (23.1) | 10 (76.9) | 6.2 (8.0, 2.0-8.0) | 0.0051 (0.0021, 0.00062-0.012) |
| 6 | 8 | 8 (100.0) | 0 (0) | 7 (87.5) | 1 (12.5) | 6.8 (7.0, 6.8-7.0) | 0.011 (0.012, 0.0063-0.014) |
| 7 | 5 | 4 (80.0) | 1 (20.0) | 0 (0) | 5 (100.0) | 4.0 (4.0, 4.0-4.0) | 0.0078 (0.0068, 0.0058-0.011) |
| 8 | 5 | 4 (80.0) | 1 (20.0) | 0 (0) | 5 (100.0) | 3.2 (3.0, 3.0-4.0) | 0.011 (0.011, 0.010-0.012) |
| 9 | 5 | 4 (80.0) | 1 (20.0) | 0 (0) | 5 (100.0) | 1.6 (1.0, 1.0-2.0) | 0.016 (0.017, 0.016-0.018) |
| 10 | 4 | 3 (75.0) | 1 (25.0) | 0 (0) | 4 (100.0) | 2.5 (2.5, 2.0-3.0) | 0.010 (0.0064, 0.0060-0.017) |
| 11 | 4 | 2 (50.0) | 2 (50.0) | 0 (0) | 4 (100.0) | 1.5 (1.0, 1.0-1.5) | 0.015 (0.014, 0.014-0.015) |
| 12 | 4 | 2 (50.0) | 2 (50.0) | 0 (0) | 4 (100.0) | 2.0 (2.0, 1.8-2.3) | 0.012 (0.014, 0.010-0.015) |
| 13-24 | 12 Triads (36 total) | 16 (44.4) | 20 (55.6) | 3 (8.3) | 33 (91.7) | — | — |
| 25-55 | 31 Dyads (62 total) | 27 (43.5) | 35 (56.5) | 3 (4.8) | 59 (95.2) | — | — |
| — | 217 Singletons | 68 (31.3) | 149 (68.7) | 0 (0) | 217 (100.0) | — | — |
| Totals | 484 | 201 (41.5) | 283(58.5) | 25 (5.2) | 459 (94.8) | — | — |

Table S3 Social-demographic characteristics of high-degree individuals (i.e., degree ≥15) within networks

| **Sample ID** | **Degree** | **Age** | **Gender** | **Ethnicity** | **Education** | **Occupation** | **Marital Status** | **Transmission Route** | **HIV Subtype** | **Recent Infection** | **History of Injection Drug Use** | **High-risk Sexual Behavior** | **Current Residence** |
| --- | --- | --- | --- | --- | --- | --- | --- | --- | --- | --- | --- | --- | --- |
| 2017QZNT243 | 47 | 46 | Male | Han | ≤6 years | Unemployee | Married | IDU | CRF08_BC | No | Yes | No | Lingshan |
| 2017QZNT210 | 35 | 37 | Male | Han | ≤6 years | Farmer | Unmarried | IDU | CRF08_BC | No | Yes | No | Lingshan |
| 2018QZNT569 | 19 | 37 | Male | Han | ≤6 years | Farmer | Unmarried | IDU | CRF08_BC | No | Yes | No | Lingshan |
| 2018QZNT622 | 18 | 38 | Male | Han | ≤6 years | Farmer | Married | IDU | CRF08_BC | No | Yes | No | Lingshan |
| 2018QZNT177 | 18 | 33 | Male | Han | ≤6 years | Farmer | Unmarried | HET | CRF01_AE | No | No | No | Lingshan |
| 2017QZNT452 | 17 | 61 | Male | Han | ≤6 years | Farmer | Married | HET | CRF07_BC | No | No | No | Qinbei |
| 2017QZNT403 | 16 | 62 | Male | Han | ≤6 years | Unemployee | Married | HET | CRF07_BC | No | No | No | Qinnan |
| 2017QZNT001 | 15 | 33 | Male | Han | 7-9 years | Worker | Married | HET | CRF01_AE | No | No | No | Pubei |
| 2017QZNT171 | 15 | 38 | Male | Han | ≤6 years | Farmer | Married | HET | CRF01_AE | No | No | No | Lingshan |
| 2017QZNT410 | 15 | 60 | Male | Han | ≤6 years | Farmer | Married | HET | CRF07_BC | No | No | Yes | Qinnan |
| 2017QZNT460 | 15 | 73 | Female | Han | ≤6 years | Unemployee | Married | HET | CRF07_BC | No | No | No | Qinbei |
| 2018QZNT052 | 15 | 75 | Male | Han | ≤6 years | Farmer | Divorced/widowed | Unknown | CRF01_AE | No | No | No | Qinbei |
| Abbreviations: IDU, intravenous drug user; HET, heterosexual; CRF, circulating recombinant form. | | | | | | | | | | | | | |

Table S4 Socio-demographic characteristics of RHI patients within networks

| **Sample ID** | **Degree** | **Age** | **Gender** | **Ethnicity** | **Education** | **Occupation** | **Marital Status** | **Transmission Route** | **HIV Subtype** | **History of Injection Drug Use** | **High-risk Sexual Behavior** | **Current Residence** |
| --- | --- | --- | --- | --- | --- | --- | --- | --- | --- | --- | --- | --- |
| 2017QZNT029 | 8 | 80 | Male | Han | ≤6 years | Farmer | Married | HET | CRF01_AE | No | Yes | Qinnan |
| 2017QZNT113 | 7 | 72 | Female | Han | ≤6 years | Farmer | Divorced/widowed | HET | CRF01_AE | No | No | Qinnan |
| 2017QZNT229 | 12 | 49 | Male | Han | 7-9 years | Farmer | Married | HET | CRF01_AE | No | Yes | Lingshan |
| 2017QZNT251 | 2 | 55 | Male | Han | 7-9 years | Farmer | Married | HET | CRF07_BC | No | No | Pubei |
| 2017QZNT464 | 1 | 43 | Male | Han | 7-9 years | Farmer | Married | HET | CRF01_AE | No | Yes | Qinbei |
| 2017QZNT501 | 14 | 35 | Male | Han | ≤6 years | Unemployee | Unmarried | HET | CRF01_AE | No | Yes | Qinnan |
| 2017QZNT514 | 7 | 66 | Male | Han | ≥10 years | Retiree | Married | HET | CRF01_AE | No | No | Qinbei |
| 2017QZNT518 | 3 | 64 | Male | Han | ≤6 years | Commercial Service | Married | HET | CRF08_BC | No | Yes | Qinnan |
| 2018QZNT115 | 2 | 68 | Male | Han | ≤6 years | Farmer | Married | HET | CRF01_AE | No | No | Qinnan |
| 2018QZNT128 | 7 | 63 | Male | Han | ≤6 years | Unemployee | Divorced/widowed | HET | CRF01_AE | No | No | Qinnan |
| 2018QZNT134 | 3 | 71 | Male | Han | ≤6 years | Unemployee | Divorced/widowed | HET | CRF08_BC | No | No | Qinnan |
| 2018QZNT188 | 12 | 48 | Female | Han | ≤6 years | Farmer | Divorced/widowed | HET | CRF01_AE | No | No | Lingshan |
| 2018QZNT200 | 10 | 51 | Female | Han | ≤6 years | Farmer | Married | HET | CRF01_AE | No | No | Lingshan |
| 2018QZNT293 | 1 | 53 | Male | Han | 7-9 years | Farmer | Married | HET | CRF01_AE | No | No | Pubei |
| 2018QZNT296 | 1 | 50 | Female | Zhuang | ≤6 years | Commercial Service | Married | HET | CRF01_AE | No | Yes | Pubei |
| 2018QZNT379 | 6 | 53 | Male | Han | 7-9 years | Farmer | Unmarried | HET | CRF01_AE | No | No | Qinnan |
| 2018QZNT422 | 7 | 55 | Female | Han | ≤6 years | Unemployee | Married | HET | CRF01_AE | No | No | Qinnan |
| 2018QZNT423 | 7 | 56 | Male | Han | 7-9 years | Unemployee | Married | HET | CRF01_AE | No | No | Qinnan |
| 2018QZNT426 | 6 | 52 | Male | Zhuang | ≤6 years | Farmer | Divorced/widowed | HET | CRF01_AE | No | Yes | Qinnan |
| 2018QZNT444 | 7 | 61 | Male | Han | 7-9 years | Unemployee | Married | HET | CRF01_AE | No | Yes | Qinnan |
| 2018QZNT479 | 1 | 41 | Female | Zhuang | ≤6 years | Farmer | Married | HET | CRF01_AE | No | No | Qinbei |
| 2018QZNT490 | 12 | 30 | Female | Zhuang | ≤6 years | Commercial Service | Unmarried | HET | CRF01_AE | No | No | Qinbei |
| 2018QZNT500 | 12 | 67 | Male | Han | ≤6 years | Farmer | Married | HET | CRF01_AE | No | No | Qinbei |
| 2018QZNT618 | 2 | 22 | Female | Han | ≤6 years | Farmer | Married | HET | CRF01_AE | No | No | Lingshan |
| 2018QZNT625 | 6 | 73 | Female | Han | ≤6 years | Farmer | Married | HET | CRF07_BC | No | No | Lingshan |
| Abbreviations: RHI, recent HIV infection; HET, heterosexual; CRF, circulating recombinant form. | | | | | | | | | | | | |

Table S5 Overview of well-supported HIV migration events across age-gender subgroups

| **HIV Subtypes** | **From** | **To** | **Mean counts** | **Bayes factor** | **Posterior probability*** | **Proportion (%)** |
| --- | --- | --- | --- | --- | --- | --- |
| **CRF01_AE** |  |  |  |  |  |  |
|  | OM | OF | 54.28 | 1123510 | 1.00 | 28.19 |
|  | OM | YF | 37.87 | 20060 | 1.00 | 19.67 |
|  | YM | YF | 18.55 | 786 | 1.00 | 9.63 |
|  | OM | YM | 81.85 | 28 | 0.93 | 42.51 |
| **CRF08_BC** |  |  |  |  |  |  |
|  | YM | OM | 21.92 | 738887 | 1.00 | 39.68 |
|  | YM | YF | 20.31 | 738887 | 1.00 | 36.77 |
|  | OM | OF | 4.45 | 341 | 0.99 | 8.06 |
|  | YM | OF | 8.56 | 305 | 0.99 | 15.50 |
| **CRF07_BC** |  |  |  |  |  |  |
|  | OM | OF | 8.35 | 182190 | 1.00 | 32.02 |
|  | OM | YM | 13.06 | 121459 | 1.00 | 50.08 |
|  | OM | YF | 4.67 | 298 | 0.99 | 17.91 |
| * indicates that only the transmission relationships with posterior probability ≥0.9 were selected. Proportions were obtained by dividing the mean counts by the sum of the mean counts for each HIV subtype dataset. Abbreviations: CRF, circulating recombinant form; OM, older male; OF, older female; YM, younger male; YF, younger female. | | | | | | |
